# Emotional Approach Coping and the Effects of Online Peer-Led Support Group Participation Among Patients With Breast Cancer: A Longitudinal Study

**DOI:** 10.2196/jmir.3517

**Published:** 2014-11-28

**Authors:** Anika Batenburg, Enny Das

**Affiliations:** ^1^VU University AmsterdamDepartment of Communication ScienceAmsterdamNetherlands; ^2^Radboud University NijmegenCentre of Language StudiesNijmegenNetherlands

**Keywords:** Internet, breast neoplasms, self-help groups, social support, online systems, emotions

## Abstract

**Background:**

Previous research on the effects of online peer support on psychological well-being of patients with cancer showed mixed findings. There is a need for longitudinal studies explaining if and when online peer-led support groups are beneficial. How patients cope with emotions that come along with the cancer diagnosis might influence effectiveness of online participation. Emotional approach coping is a construct encompassing the intentional use of emotional processing and emotional expression in efforts to manage adverse circumstances.

**Objective:**

In this longitudinal study, we hypothesize that mixed findings in previous research are partly caused by individual differences in coping with emotions, which may moderate the effects of online support group participation on patients’ well-being.

**Methods:**

A total of 133 Dutch patients with breast cancer filled out a baseline (T0) and a follow-up (T1, 6 months later) questionnaire assessing intensity of online participation within the online support community, emotional approach coping (ie, actively processing and expressing emotions), and psychological well-being (depression, emotional well-being, and breast cancer–related concerns). There were 109 patients who visited an online support community at both points in time. Repeated measures ANOVAs assessed change in well-being over time.

**Results:**

Results showed 3-way interactions of time, online intensity of participation, and emotional approach coping on emotional well-being (*F*_1,89_=4.232, *P*=.04, η^2^_ρ_=.045) and depression (*F*_1,88_=8.167, *P*=.005, η^2^_ρ_=.085). Online support group participation increased emotional well-being over time for patients who scored low on emotional approach coping at T0, provided that they were highly active online. Patients who were highly active online with a high score on emotional approach coping reported no change in sense of well-being, but showed the highest score on well-being overall. Participating less frequently online was only beneficial for patients who scored high on emotional approach coping, showing an increase in well-being over time. Patients participating less frequently and with a low score on emotional approach coping reported no significant change in well-being over time.

**Conclusions:**

This study extends previous findings on the effects of online peer support in two ways: by testing changes in well-being as a function of intensity of online support group participation and by examining the role of individual differences in emotional coping styles. Findings showed no negative effects of intense support group participation. Participating frequently online was especially helpful for patients who approach their emotions less actively; their emotional well-being increased over time. In contrast, frequent online users who actively approach their emotions experienced no change in well-being, reporting highest levels of well-being overall. For patients who participate less intensively within the support community, coping style seems to outweigh effects of online participation; over time, patients who actively approached emotions experienced an increase in psychological well-being, whereas patients with a low score on emotional approach coping reported no change in depression and emotional well-being.

## Introduction

An increasing amount of individuals are diagnosed with breast cancer, and this number is expected to grow over the coming years due to early diagnosis, average increase in human life expectancy, and more survivors due to improved treatment [1]. A growing number of patients turn to the Internet to search for illness-related information and seek support among peers. Patients with breast cancer are among the most active online seekers compared to other patient groups [2]. Therefore, the question if such online communities are beneficial for patients becomes increasingly important.

The lack of longitudinal studies testing causal effects of online peer-led support group participation is most likely due to its uncontrolled setting. Testing effects is difficult because patients participate anonymously and autonomously—they determine when and how much they want to participate. Participants become members at different time points, frequency and length of visits vary among patients, and some patients are active posters whereas others only read messages from others (ie, lurkers) [3]. Perhaps as a result, studies covering peer-led support communities are often descriptive in nature (ie, interview studies, content analyses, cross-sectional surveys). These studies point to the presence of empowering and therapeutic processes, such as emotional and informational support, recognition, and understanding, but also disempowering processes such as being confronted with negative sides of the disease and complainers [4-8]. However, effects are rarely tested although these peer-led platforms are easily accessible and common online.

Current outcome studies on online peer support mostly concern online interventions set up by health professionals, showing positive effects, such as decreased depression, posttraumatic stress, and perceived stress [9-12]. However, it has been discussed that these online interventions often include several other therapeutic aspects besides peer support (eg, decision-making tools, skill training, or professional moderation). It is unclear if improvements among patients are specifically caused by the sheer support from peers or by other aspects of the intervention [13]. Only a few studies empirically tested the effects of online peer-led support communities. For example, Lieberman and Goldstein [14] showed a positive change in emotional well-being, depression, and posttraumatic growth. However, other studies reported no significant major effects [15,16] or negative effects [17]. If we assume that null findings may not always get published due to publication bias [13], the meager evidence for a direct relationship between online peer support and well-being may even be an overrepresentation. This requires more studies testing when and why online peer support is beneficial. Therefore, the first goal of the present research was to provide a robust test of changes in patients’ psychological well-being over time due to patients’ intensity of online peer-led support group participation.

We put forward that patients’ intensity of online participation is important to assess when we aim to test the effectiveness of online peer support group participation because differences in patients’ online behavior might affect health outcomes. For example, a cross-sectional study showed that posters felt that they received more benefits (ie, emotional support, helping others, and emotional expression) from online communities than lurkers, and only for posters were higher levels of emotional support/helper therapy and advice related to lower levels of anxiety [3]. In this longitudinal study, we aim to reveal if patients who are more active within a peer-led support community (in terms of frequency and length of visits and amount of posts) benefit more in terms of health outcomes than patients who are less active online.

Apart from intensity of online participation, patients also differ on aspects outside the online environment, which may affect online behavior and effectiveness of online support group participation as well. For example, recent studies suggest that individual differences in health self-efficacy [18], emotional communication competence [19], and differences in coping with emotions [20] might be moderating factors. In this study, we focus on patients’ level of emotional approach coping (ie, actively processing and expressing emotions). Evidence from related fields such as clinical psychology shows substantial differences between patients in coping with cancer-related emotions, which significantly affect ones’ well-being. Because online support platforms are merely used to share illness-related experiences (ie, patients write about their experiences and related thoughts and emotions), variations in patients’ emotional coping styles might be one of the reasons why a direct relation between online participation and well-being is not always found. Studies showed that actively coping with emotions (ie, recognizing and feeling the meaning of losses) [21] is often related to better well-being. For example, actively approaching emotions is related to decreased depressive symptoms, distress, increased vigor, improved perceived health status, and fewer medical appointments [22-25]. Hence, how patients cope with emotions that come along with the cancer diagnosis might influence effectiveness of online participation as well.

Our presumption that the relationship between intensity of online participation and well-being might be moderated by patients’ emotional coping style is substantiated by 2 studies on writing style within online peer-led support communities and a cross-sectional study on emotional approach coping. The first 2 studies showed that the use of words related to expression of emotions and to learning and understanding was related to changes in well-being [26,27]. However, whether word use caused changes in well-being or was a reflection of well-being remains unclear. Nevertheless, these findings show that patients differ in how they deal with illness and emotions in online environments. Another recent cross-sectional study showed that emotional approach coping was especially relevant for patients who participated frequently within an online peer-led support group (ie, for patients who visit the online community and post messages relatively often). Patients who actively dealt with their emotions and frequently participated online reported higher psychological well-being than patients who were frequent users but approached their emotions less actively. No difference in well-being was found for patients who participated less often within the online support group [20].

Although these findings underscore the importance of individual differences in emotional approach coping when assessing effects of online support group participation, they provide no conclusive evidence regarding causal patterns. Therefore, we aimed to extend these cross-sectional findings by tracking patients over an extended period of time to further assess individual emotional coping differences. In-line with previous cross-sectional findings, we expected that patients who actively deal with emotions may benefit especially from online support groups. Because online support communities often confront patients with emotionally distressing content from peers [28], patients who participate intensely but cope with emotions less actively, might experience additional stress ending up in a downward spiral. However, one might also argue that over time online support and the recognition patients find in stories from others might be especially helpful for patients with more repressive coping styles because they need it most.

In this research project, we conducted a 2-wave longitudinal study among Dutch patients with breast cancer participating in online peer-led support communities, in which we assessed individual differences in emotional coping style. Specifically, we assessed patients’ intensity of online support group participation, emotional approach coping [25], and 3 measures of well-being that are generally associated with breast cancer diagnosis at 2 points in time: emotional well-being [29], depression [30], and breast cancer-related concerns [31]. We included potential covariates (ie, factors often associated with the psychological well-being of patients with breast cancer), such as social support from family and friends [32,33], disease status, and received professional psychological help. Based on previous findings regarding emotional approach coping, we propose an interaction effect of emotional coping style and the intensity of patients’ online participation. With our longitudinal approach, we aim to reveal the long-term effects of this interaction on patients’ psychological well-being.

## Methods

### Participants and Procedure

We searched the Internet with Google to identify all online support communities for patients with breast cancer in the Netherlands. Criteria for inclusion were (1) the website was in the Dutch language, (2) the website was purely designed as 24-hour available message boards or part of the website was designed as a 24-hour available message board, and (3) the discussion board was still active (new messages were posted within the past month). With approval of the website owners, we posted a request to participate in an online survey about Internet use of patients with breast cancer on 7 support websites. Participants filled out a baseline (T0) survey in June 2011, including demographics, disease status, the intensity of online support group participation, and psychological well-being (the specific measurements used in this study are described subsequently). After 6 months, we sent this group of patients a follow-up (T1) questionnaire (December 2011) to reassess their psychological well-being and intensity of online support group participation.

This survey was part of a more extensive research project on online peer support among Dutch patients with breast cancer. The research was carried out in accordance with the American Psychological Association’s ethics guidelines [34] and complied with European Union legislation [35] and Dutch legislation [36] on data protection. All procedures were approved by the Department of Communication Science at VU University Amsterdam.

The introduction page of the survey included the length and purpose of the survey, contact information of the investigator, and ensured anonymity. A sample of 134 Dutch breast cancer survivors filled out both questionnaires. Because this sample included 133 females and only 1 male, we decided to exclude this male from the data analyses to keep a homogeneous group. Response rates are unknown because we had no access to page views of the participating websites. The online survey tool tracked Internet protocol (IP) addresses to prevent users from retaking the survey. Responses to questions were obligatory, but participants were provided with an “I don’t know” or “not applicable” option.

### Measurements

#### Demographics, Illness Characteristics, and Control Variables

The T0 baseline questionnaire included questions on patients’ age, gender, education level, and working status (ie, if patients were currently working). We measured current disease status (ie, if cancer cells were currently detected in the patient’s body or not), the number of medical appointments in the past 3 months regarding breast cancer, and if patients were under treatment at the moment. Because social support from resources other than online peers may affect psychological well-being, we asked if patients received any psychological help from a professional and assessed the social support they received from their friends and family based on the 6 social well-being items from the Functional Assessment of Cancer Therapy-Breast (FACT-B) [29]. Items referring to support from friends were adjusted into items that clearly referred to offline friends. Respondents rated on a 5-point scale if the statements applied to them, ranging from “not at all” to “totally” (Cronbach alpha=.753).

#### Intensity of Online Support Group Participation

At both T0 and T1, patients’ intensity of online support group participation was assessed by 4 different questions regarding frequency of visits, average length of visits, contribution (ie, reading, responding, starting new topics or questions), and frequency of posts in the last 4 weeks [20,37]. Frequency of visits was assessed on a 7-point scale; the other items were assessed on a 4-point scale. To merge these different scales into 1 index, all items were transformed into *z* scores. The scale was internally consistent at T0 (Cronbach alpha=.799) and T1 (Cronbach alpha=.796). See Multimedia Appendix 1 for the specific items.

#### Emotional Approach Coping

At T0 the emotional approach coping scale [25] was used to measure participants’ coping style concerning emotions, including 4 items referring to emotional processing (eg, “I realize that my feelings are justified and important”) and 4 items regarding emotional expression (eg, “I take the time to express my emotions”). Participants rated on a 4-point scale if the statements applied to them. The mean scores (emotional processing: mean 2.85, SD 0.63; emotional expression: mean 2.85, SD 0.57) were comparable to those of a sample of patients with breast cancer from a previous study (emotional processing: mean 3.00, SD 0.72; emotional expression: mean 2.95, SD 0.84 [23]) and a group of healthy women (emotional processing: mean 2.85, SD 0.63; emotional expression: mean 2.79, SD 0.73 [25]).

Because factor analyses showed that all factors loaded between 0.58 and 0.86 on 1 component and explained 44% of the variance, we created 1 index for emotional approach coping with all 8 items. Ratings were summed and averaged across items (Cronbach alpha=.864).

#### Psychological Well-being

At both T0 and T1, we assessed psychological well-being with 3 different concepts: depression, breast cancer-related concerns, and emotional well-being. Depression was measured with the CES-D10 [30]. The scale consisted of 10 items (eg, “I felt that everything I did took me quite some effort”). Participants rated on a 4-point scale if the statements applied to them the past week from “less than 1 day” to “5 to 7 days.” The scale was internally consistent in both questionnaires (T0: Cronbach alpha=.740; T1: Cronbach alpha=.815), but was positively skewed at T1. A log transformation was performed for depression T0 and T1 to meet the assumptions of multiple regression analysis [38]. Breast cancer-related concerns (Profile of Concerns about Breast cancer [31]) were measured with an index of 28 items assessed on a 5-point scale (eg, “As you think about your illness, how much are you concerned that chemotherapy or radiation therapy will damage your body in some way?”). The index showed consistency (T0: Cronbach alpha=.909; T1: Cronbach alpha=.918). Emotional well-being was measured according to 6 items from the FACT-B on a 5-point scale (eg, “I’m proud of how I am coping with my illness” [29] and showed scale consistency at T0 (Cronbach alpha=.821) and T1 (Cronbach alpha=.876).

### Analyses

We conducted repeated measures ANOVAs on the 3 measures of psychological well-being with intensity of online participation at T0 (-1 SD vs +1 SD) and emotional approach coping (-1 SD vs +1 SD) at T0 as between-subjects factors and time (T0 vs T1) as a within-subjects factor (see [39] for this specific regression analysis). This estimation procedure allows tests of differences between participants with low vs high levels of online support group participation and participants with low vs high levels of emotional approach coping without conducting a median split, thus retaining all observations in the analysis [40]. We added the intensity of online participation at T1 as covariate in our model to control for changes in online participation over time. In addition, we also included disease status at T1 as control variable into the model. Finally, all other variables that correlated significantly with the independent and dependent variables (ie, intensity of online participation T0, emotional approach coping T0 or psychological well-being T0 and T1 were entered into the model as covariates (see Results).

## Results

### Sample Characteristics

Table 1 presents the patients’ characteristics. Our sample of 133 patients with breast cancer included women with a mean age of 48.44 years (SD 8.60). Most patients had an average to high level of education (86/132, 65.2%), and more than half (79/133, 59.4%) were (still) actively performing their job. Of the sample, 67.5% reported that no cancer cells were detected at the moment. Slightly more than half of the participants (68/133, 51.1%) were under treatment, the other half (65/133, 48.9%) were not under treatment or only monitored by a physician at the moment. The average number of breast cancer–related medical appointments in the previous 3 months before participating in this study was 3.6 appointments. Less than half of the participants received psychological guidance during the period of illness (57/132, 43.2%).

**Table 1:** Demographics and health characteristics of participants.

Characteristic			Participants
**Age (n=133)**			
	Mean (SD)		48.44 (8.60)
	Range		23-67
**Highest education level** ^a^ **(n=132), n (%)**			
	Primary school		4 (3.0)
	**Secondary school**		
		Low (junior general secondary school)	20 (15.2)
		Middle (senior general secondary school)	12 (9.1)
		High (pre-university)	1 (0.8)
	**Vocational school**		
		Low^b^ (LBO/LTS)	9 (6.8)
		Middle (MBO)	36 (27.3)
		High (HBO) (Bachelor’s degree)	41 (31.1)
		Scientific degree (Bachelor/Master’s degree)	9 (6.8)
**Working status (n=133), n (%)**			
	Not working		53 (40.2)
	Working		79 (59.8)
**Disease status (n=123), n (%)**			
	No cancer cells		83 (67.5)
	Cancer cells		40 (32.5)
**Under treatment (n=133), n (%)**			
	Yes		68 (51.1)
	No		65 (48.9)
**Number of cancer-related medical appointments in the past 3 months**			
	Mean (SD)		3.61 (6.34)
	Range		0-40
**Psychological help during period of illness (n=132), n (%)**			
	Yes		57 (43.2)
	No		75 (56.8)

^a^ Levels within the Dutch education system: education is divided over 3 schools for different age groups, which are divided in streams for different educational levels.

^b^ LBO/LTS (ie, lowest level of vocational school) existed until 1992.

### Correlations

We first ran a correlation matrix (Tables 2 and 3) to assess associations between independent and dependent variables, and to detect potential covariates. No direct correlations between the intensity of online participation at T0, emotional approach coping at T0, breast cancer–related concerns (at both T0 and T1), emotional well-being (at both T0 and T1), and depression (at both T0 and T1) were found. Because of significant correlations with the independent or dependent measures, we added age, education level, disease status, offline social support, and psychological help from a professional into our models as covariates.

**Table 2:** Means, standard deviations, and intercorrelations of independent variables, covariates, and dependent variables (part 1).

Variables		N	Mean (SD)	1	2	3	4	5	6	7
1	Intensity of online participation T0^a^	125	0.00 (0.78)	–						
2	Intensity of online participation T1^a^	113	–0.01 (0.79)	.70^c^						
3	Emotional approach coping	132	2.86 (0.50)	.04	–.03					
4	Age	133	48.44 (8.60)	–.23^c^	–.21^d^	–.14				
5	Education	132	6.38 (1.95)	–.06	–.07	.06	–.21^d^			
6	Working status^b^	132	0.60 (0.49)	–.06	–.02	–.05	–.21^d^	.37^c^		
7	Offline social support	133	3.80 (0.62)	.04	.01	.04	.04	.24^c^	.14	
8	Psychological help^b^	132	0.43 (0.50)	–.01	–.01	.12	–.45^c^	.08	.15	–.23^c^
9	Disease status T1^b^	123	0.33 (0.47)	.03	.11	.03	–.07	–.09	–.13	–.06
10	Under treatment^b^	133	0.51 (0.50)	.13	.17	.03	–.10	–.02	–.07	.09
11	Medical appointments	132	3.61 (6.34)	.15	.22^d^	.12	.02	–.11	–.20^d^	.09
12	Depression T0 (log)	132	0.25 (0.11)	–.04	–.04	.01	–.13	–.20^d^	–.13	–.41^c^
13	Depression T1 (log)	133	0.22 (0.13)	.05	.09	–.16	–.12	–.23^c^	–.16	–.37^c^
14	Emotional well-being T0	133	3.53 (0.80)	–.04	–.04	–.08	.12	.18^d^	.20^d^	.43^c^
15	Emotional well-being T1	133	3.70 (0.90)	–.08	–.12	.11	.13	.18^d^	.12	.37^c^
16	Breast cancer–related concerns T0	133	2.61 (0.60)	.09	.13	.09	–.18^d^	–.23^c^	–.24^c^	–.46^c^
17	Breast cancer–related concerns T1	133	2.52 (0.63)	–.03	–.01	–.03	–.10	–.26^c^	–.26^c^	–.41^c^

^a^ Standardized into Z scores.

^b^ Coded 0=no, 1=yes.

^c^ Correlations significant at the .01 level.

^d^ Correlations significant at the .05 level.

**Table 3:** Means, standard deviations, and intercorrelations of independent variables, covariates, and dependent variables (part 2).

Variables		8	9	10	11	12	13	14	15	16
9	Disease status T1^a^	.00								
10	Under treatment^a^	–.12	.28^b^							
11	Medical appointments	.05	.17	.10						
12	Depression T0 (log)	.34^b^	–.08	–.12	.04					
13	Depression T1 (log)	.30^b^	.14	–.01	.09	.54^b^				
14	Emotional well-being T0	–.29^b^	–.10	.13	–.11	–.57^b^	–.48^b^			
15	Emotional well-being T1	–.27^b^	–.10	.07	–.05	–.39^b^	–.62^b^	.65^b^		
16	Breast cancer–related concerns T0	.32^b^	.01	–.09	.001	.51^b^	.50^b^	–.60^b^	–.42^b^	
17	Breast cancer–related concerns T1	.29^b^	.02	–.01	–.003	.50^b^	.57^b^	–.52^b^	–.55^b^	.65^b^

^a^ Coded 0=no, 1=yes.

^b^ Correlations significant at the .01 level.

### Effects Testing

From the 133 patients, 125 patients reported they were visiting an online breast cancer support community at T0; 8 patients were not. The study participants only had access to the questionnaire by visiting the online support community; therefore, they might have misunderstood our question. Furthermore, because these 8 patients claimed they did not visit an online support group, they also did not fill out our questions on intensity of participation and were excluded from data analyses.

At T1, 113 patients claimed they were visiting an online support group. From T0 to T1, 16 participants stopped visiting the online support community and 4 participants started visiting an online community after the first survey. To measure the change in psychological well-being over time caused by the intensity of online support group participation, we only included the patients that were visiting an online support community at both time points (n=109). To prevent our study from unnecessary data loss, 1 of the reviewers recommended adding an additional lowest category to the items measuring intensity of online participation T1 to include the forum users who stopped visiting the forum from T0 to T1. However, this new variable violated the normal distribution norms for regression analyses. Therefore, we could not include these support group dropouts for data analyses. Nevertheless, when including this new variable in the analyses, the 3-way interactions were still significant (depression: *F*_1,105_=6.360, *P*=.01; emotional well-being: *F*_1,105_=4.232, *P*=.04).

Of the 109 participants who visited an online community at T0 and T1, 9 participants were not aware of their disease status, 1 participant did not report her level of education (see Table 1), and the level of depression at T0 was missing for 1 participant. Because we included these variables as covariates, SPSS excluded these participants from the ANOVA analyses.

Table 4 shows the within-subjects ANOVAs, indicating a 3-way interaction of time, intensity of online participation at T0, and emotional coping T0 on depression and emotional well-being. No 3-way interaction effect on breast cancer–related concerns was found.

**Table 4:** Repeated measures ANOVAs on well-being measures at T0 and T1.

Independent variables		Depression (n=98)			Emotional well-being (n=99)			Breast cancer concerns (n=99)		
		*F*_1,88_	*P*	η^2^_ρ_	*F*_1,89_	*P*	η^2^_ρ_	*F*_1,89_	*P*	η^2^_ρ_
**Between-subjects effects, (mean T0 and T1)**										
	Intensity of online participation T0	0.429	.51	.005	1.197	.28	.013	0.343	.56	.004
	Emotional approach coping	4.620	.03	.050	1.285	.26	.014	0.017	.90	.000
	Education	6.032	.02	.064	3.278	.07	.036	4.295	.04	.046
	Offline support	7.358	.008	.077	10.684	.002	.107	17.834	<.001	.167
	Psychological help	14.095	<.001	.138	8.174	.005	.084	7.033	.009	.073
	Age	0.181	.67	.002	0.005	.95	.000	0.814	.37	.009
	Intensity of online participation T1	0.564	.45	.006	3.428	.07	.037	1.295	.26	.014
	Disease status	0.103	.75	.001	0.288	.59	.003	0.470	.50	.005
	Intensity of online participation T0 × emotional approach coping	1.738	.19	.019	1.568	.21	.017	0.049	.83	.001
**Within-subjects effects**										
	Time	0.773	.38	.009	1.041	.31	.012	2.355	.13	.026
	Time × intensity of online participation T0	0.104	.75	.001	0.102	.75	.001	0.272	.60	.003
	Time × emotional approach coping	1.185	.28	.013	1.166	.28	.013	0.612	.44	.007
	Time × education	0.343	.56	.004	0.104	.75	.001	0.031	.86	.000
	Time × offline support	0.107	.11	.745	0.664	.42	.007	2.552	.11	.028
	Time × psychological help	0.282	.28	.597	0.068	.80	.001	0.334	.57	.004
	Time × age	0.663	.42	.007	0.027	.87	.000	0.409	.52	.005
	Time × intensity of online participation T1	1.231	.27	.014	0.711	.40	.008	2.352	.13	.026
	Time × disease status	9.210	.003	.095	0.084	.77	.001	0.489	.49	.005
	Time × intensity of online participation T0 × emotional approach coping	8.167	.005	.085	4.232	.04	.045	2.450	.12	.027

### Depression

**Figure 1:**
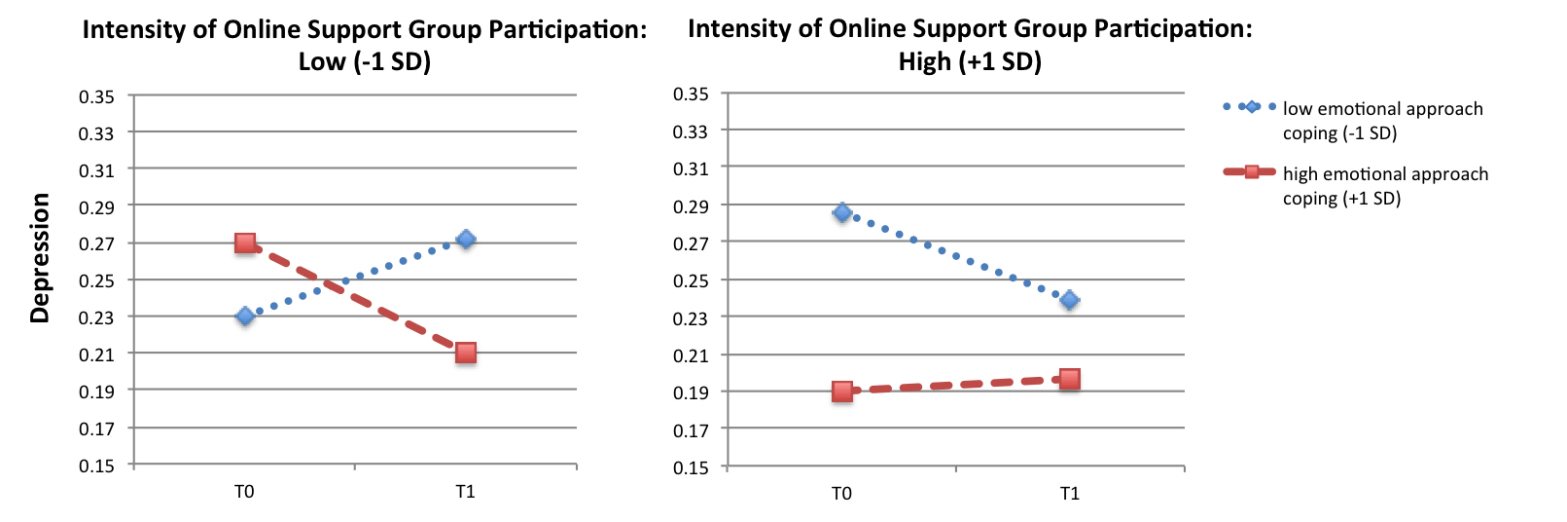
Depression over time (T0, T1) as a function of intensity of online participation at T0 (low vs high) and emotional approach coping (low vs high).

No main effect of time on depression was found; overall depression did not change significantly from T0 to T1 (see Table 4). Concerning covariates, only disease status caused a change in depression. However, education, offline social support, and professional psychological help were related to the average score of depression at T0 and T1. No 2-way interactions of time and intensity on online participation, or time and emotional approach coping on depression were found. However, a 3-way interaction effect of time, the intensity of online participation, and emotional approach coping was found. Pairwise comparisons revealed that patients who scored low on online participation and high on emotional approach coping showed a significant decrease in depression over time (mean difference 0.060, SE 0.027, *P*=.03) (see Figure 1). In addition, online frequent users who scored high on emotional approach coping did not show a change in depression over time (*P*=.80), but showed lowest overall levels of depression at both points in time (Figure 1).

### Emotional Well-Being

**Figure 2:**
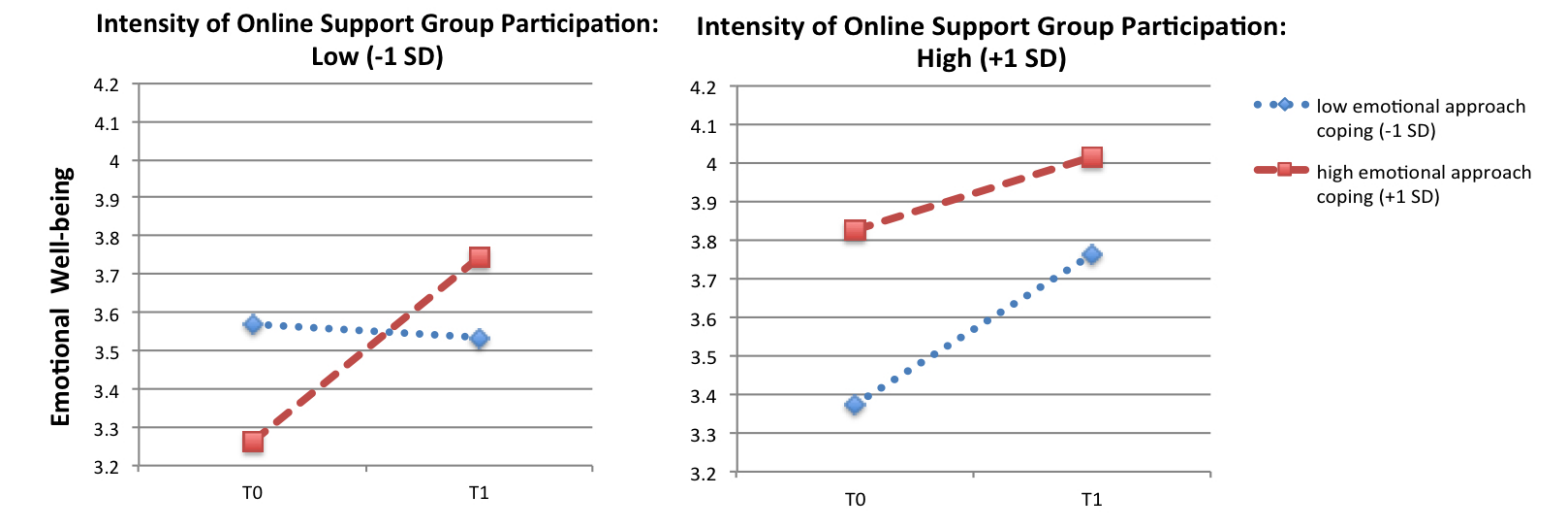
Emotional well-being over time (T0, T1) as a function of intensity of online participation at T0 (low vs high) and emotional approach coping (low vs high).

Table 4 shows no main effect of time on emotional well-being; overall emotional well-being did not change significantly from T0 to T1. No covariates were related to changes in well-being, although offline social support and receiving professional psychological help were related to the average score of emotional well-being. No 2-way interaction of time and intensity of online participation, or time and emotional approach coping were found. However, a significant 3-way interaction of time, online intensity of participation, and emotional approach coping on emotional well-being was found. Pairwise comparisons showed that emotional well-being increased over time for patients who scored low on intensity of participation but high on emotional approach coping (mean difference 0.479, SE 0.169, *P*=.006; see Figure 2). No significant change in well-being was observed among patients who scored low on online participation and low on emotional approach coping (*P*=.86). In addition, for patients who scored high on online participation and low on emotional approach coping, emotional well-being increased over time (mean difference 0.391, SE 0.181, *P*=.03; see Figure 2). For frequent online users who scored high on emotional approach coping, no significant change in well-being was found, but showed highest levels of well-being at both points in time (*P*=.22).

### Breast Cancer–Related Concerns

Only education, psychological help from a professional, and offline social support were related to the average score of breast cancer–related concerns. No other main or interaction effects were found.

## Discussion

### Principal Findings

This 2-wave longitudinal study extends previous findings on the effects of online peer support in 2 ways: by testing changes in well-being as a function of intensity of online support group participation and by examining the role of individual differences in emotional coping styles. Findings showed that participating frequently online was especially helpful for patients who approach their emotions less actively; their emotional well-being increased over time. In contrast, frequent online users who actively approach their emotions experienced no change in well-being, reporting highest levels of well-being overall. For patients who participate less intensively within the support community, coping style seems to outweigh effects of online participation; over time, patients who actively approached emotions experienced an increase in psychological well-being, whereas patients with a low score on emotional approach coping reported no change in depression and emotional well-being.

This study was stimulated by findings from a previous cross-sectional study that showed that approaching one’s emotions was especially relevant for patients who were highly active within an online support community [20]. Specifically, frequent users with a low score on emotional processing and expression reported significantly lower psychological well-being than equally active patients but with a high score on emotional processing and expression. In contrast, current longitudinal findings showed that especially the frequent users who approach emotions less actively benefit from intense online participation; they experienced an increase in emotional well-being over time. An explanation might be that frequent online participation compensates for the negative effects of not approaching emotions. Patients may benefit from several therapeutic processes within the online support group, such as the support from peers, empowerment by the provision of relevant illness-related information, and recognition in stories from others. These helpful processes might compensate for negative effects of not actively approaching emotions and cause a positive change in patients’ well-being.

These results reconcile previous null findings of support group participation on well-being. We found no main effect of the level of online participation on changes in well-being; we only found interactions of online participation with patients’ emotional coping style. This underscores the importance of considering individual differences in dealing with illness when examining health outcomes of online support communities. Although we found no negative effects for highly frequent users, researchers should be careful not to treat an online support community as a “one-cure-fits-all” solution because, apparently, they are not.

The present findings also showed the importance of other factors outside the online environment. We found, for example, correlations between patients’ well-being and offline social support and professional psychological help. Patients who felt supported by family and friends reported a higher well-being than patients’ feeling less supported, and patients receiving professional help reported a lower well-being. Although such covariates showed no influence on well-being change (except for disease status), we should be careful not to overestimate the effects of online peer support. Since no direct relation between online intensity of participation and well-being was found, such “offline” factors seem to have a stronger influence on patients’ well-being than the level of online participation within an online peer support group. Online peer support might contribute to patients’ well-being, but it probably does not compensate for the potential negative effects of other important factors, such as a lack of support from relatives and deteriorated health status.

Regarding practical implications, with caution we could state that patients might be encouraged to participate actively in online peer communities. Although we cannot compare the current participating group with patients who decide not to visit online support communities, for the present sample frequent participation was beneficial to patients’ well-being in the long run, regardless of their coping style, and did not cause harm to any patient group. Especially patients who occasionally visit an online community and approach their emotions less actively should be stimulated to participate more frequently because they seem to benefit most from frequent participation.

### Limitations and Future Research

No interaction effects of our key independent variables on breast cancer–related concerns were found. One explanation might be that breast cancer–related concerns are no accurate measure for well-being. Although the other psychological well-being measures were related to breast cancer–related concerns, concerns might be less appropriate to use as an outcome measure because concerns might intensify online participation. To be more specific, concerns may induce the need for online information and support, and for some patients online peer support, in turn, increases well-being over time. Another explanation is that concerns depend mostly on disease factors rather than online peer support. For example, current treatment, physical well-being, or recurrence might induce concerns. Such factors might intensify concerns at different (shorter) time points; therefore, concerns may fluctuate more intensively over time than more “stable” measures of well-being, such as depression. Future studies should include a more detailed assessment of the disease process patients are in and the number of months they have been participating in an online forum to test this assumption.

In this study, we measured emotional approach coping only in the baseline questionnaire because coping style is merely viewed as an aspect of ones’ personality that is rather stable over time and, therefore, often measured just once in longitudinal studies (eg, [23]). However, studies with a cognitive-behavioral approach suggest that specific treatment has the potential to teach patients certain coping skills [41]. Although peer-led support communities do not include professional guidance, it might still be possible that online conversations stimulate approaching emotions, which has a positive influence on their well-being. From a modeling or skills perspective [42], frequent participation in online support communities may help patients with more repressive coping styles to learn over time how to approach illness-related emotions. In future studies, we recommend to reassess coping style to test if patients learn to cope with emotions due to online participation.

Examining peer-led online communities is difficult because of its’ anonymity and “fluidness” (ie, patients come and go whenever they like). Because participating patients were anonymous, there is no record of what participants did before T0, no information on dropouts, and the findings rely on self-report (ie, there is no actual information on what they precisely did online). We have no information on participants’ starting date of online participation. Possibly, advantages of online participation might particularly occur for patients who just started to participate online, for example because they are recently diagnosed and especially in need of support. It is possible that changes in well-being might be less pronounced in patients who are part of the community for a longer period. Future research should examine this possibility.

Furthermore, we may have encountered selection bias. Patients who were willing to participate in the current study might also be more involved in online support and, therefore, already behave differently than patients less active online. With respect to the current limitations, in future research it would be interesting to track patients’ actual online behavior. Although it is rather difficult when it comes to anonymous peer-led online communities, investigating patients’ online writings and connecting these findings to measures of well-being and individual characteristics would provide a better understanding of online processes and its effects. For instance, it would be interesting to see if certain coping skills reflect online writing style.

Although this is one of the rare attempts to investigate longitudinal effects of peer-led support communities, tracking patients for an even longer period with more points of measurement might answer more questions on this topic. For example, online participation may not only influence well-being, patients’ level of well-being might also incite online participation. For instance, patients’ negative experiences lower well-being, which may stimulate online support seeking and, in turn, online interactions with peers helping patients to cope with the current situation, which in turn increases well-being.

Furthermore, in this study we investigated emotional approach coping. However, other individual differences might be of interest as well. For instance, are patients satisfied with the support they receive online and how does this affect well-being? Or how do patients compare their own situation to the condition of peers and how does this affect them? Future research should address other underlying psychological mechanism that could influence the effects of online support group participation.

### Conclusions

To our knowledge, this study is one of the first studies to examine the effects of online peer-led support group participation, taking into account individual differences in emotional coping style. Over time, frequent participation in an online peer support community increased emotional well-being, in particular for patients low on emotional approach coping. Findings suggest that intense support group participation may be especially helpful for patients who approach emotions less actively.

## Multimedia Appendix 1

Scale construction: intensity of online support group participation.

## Abbreviations

IP: Internet protocol
