# Supplementary material for: Emotional Approach Coping and the Effects of Online Peer-Led Support Group Participation Among Patients With Breast Cancer: A Longitudinal Study
Source: J Med Internet Res. 2014 Nov 28;16(11):e256. doi: 10.2196/jmir.3517 (PMC4260065; doi:10.2196/jmir.3517)
Supplement: Supplementary file 1 [file jmir_v16i11e256_app1.pdf]

Multimedia Appendix 1: Scale Construction - Intensity of Online Support Group Participation

The next questions refer to how frequently and intense you participate on the online forum.

If you think about the last four weeks…

Frequency of visits
How often did you visit the online forum?

- Less than once a month
- Approximately once a month
- Multiple times per month
- Approximately once a week
- Multiple times per week
- Approximately once a day
- Multiple times per day

Average length of visits
What was the average length per visit?

- Less than 10 minutes
- 10 to 30 minutes
- 30 minutes to one hour
- More than one hour

Forum contribution
Did you contribute to the online forum?

- I only read posts from others
- I reacted on (a) post(s) of someone else
- I started a new topic or asked a question
- I both started a new topic/asked a question AND I reacted on (a) post(s) of another

Frequency of posts
How many messages* did you post?
(*initial posts and reactions to questions or stories from others)

- None
- One per week or less
- Multiple posts per week, but not every day
- Every day one post or more
